# Nitric Oxide-Dependent Posttranslational Modification in Plants: An Update

**DOI:** 10.3390/ijms131115193

**Published:** 2012-11-16

**Authors:** Jeremy Astier, Christian Lindermayr

**Affiliations:** Institute of Biochemical Plant Pathology, Helmholtz Zentrum München, Ingolstädter Landstr. 1, 85764 Neuherberg, Germany; E-Mails: jeremy.astier@helmholtz-muenchen.de (J.A.); lindermayr@helmholtz-muenchen.de (C.L.); Tel.: +49-89-3187-2129 (J.A.); +49-89-3187-2285 (C.L.); Fax: +49-89-3187-3383 (J.A.); +49-89-3187-3383 (C.L.)

**Keywords:** metal nitrosylation, *S*-nitrosylation, tyrosine nitration, nitric oxide, posttranslational modification, plants

## Abstract

Nitric oxide (NO) has been demonstrated as an essential regulator of several physiological processes in plants. The understanding of the molecular mechanism underlying its critical role constitutes a major field of research. NO can exert its biological function through different ways, such as the modulation of gene expression, the mobilization of second messengers, or interplays with protein kinases. Besides this signaling events, NO can be responsible of the posttranslational modifications (PTM) of target proteins. Several modifications have been identified so far, whereas metal nitrosylation, the tyrosine nitration and the S-nitrosylation can be considered as the main ones. Recent data demonstrate that these PTM are involved in the control of a wide range of physiological processes in plants, such as the plant immune system. However, a great deal of effort is still necessary to pinpoint the role of each PTM in plant physiology. Taken together, these new advances in proteomic research provide a better comprehension of the role of NO in plant signaling.

## 1. Introduction

Nitric oxide (NO) is a ubiquitous diatomic gas that has been described as an important regulator of a wide range of physiological processes in animal models [1]. NO production is not restricted to animal cells, and several studies have shown that it takes place in other kingdoms, such as plant organisms [2]. Several lines of evidence indicate that NO can be synthesized in plants from nitrite, polyamines and L-arginine, through nonenzymatic or enzymatic mechanisms [3,4]. However, with the exception of nitrate reductase, the corresponding enzymes are yet to be identified, and understanding NO generation in plants remains an important challenge [4,5]. Nevertheless, the functions of NO in plants have been widely studied over the past decades and a significant amount of evidence demonstrated the involvement of NO in the regulation of several biological processes, including hormonal signaling, root growth, stomatal closing, iron homeostasis, germination, or pollen tube growth. Moreover, NO participates in the establishment of adaptive responses against biotic and abiotic stresses in plants [2,6–8].

Although the described NO functions in plants have been increasing over the last years, the precise molecular mechanisms underlying its physiological roles are still poorly understood. Some works about NO way of action in plants demonstrated that artificially generated as well as endogenously produced NO can modulate several gene expressions, involved in stress responses, hormonal signaling or primary metabolism [4,9–11]. In addition, NO has been demonstrated to impact cGMP, Ca^2+^, protein kinase, ROS or phytohormones signaling in plants [2,12–14]. Moreover NO can directly modify target proteins by posttranslational modification (PTM), three of them being the major NO-dependent PTM. The first one concerns the interaction of NO moiety with metalloproteins in a so-called metal nitrosylation. The second refers to the modification of tyrosine residues of proteins by NO, resulting to the formation of 3-nitrotyrosine. The last one concerns the formation of a nitrosothiol group on cysteine residues of target proteins, in a reaction called *S*-nitrosylation. Under distinct conditions, other NO-dependent modifications, such as *S*-glutathionylation or formation of disulfide bridges, are also observed [15–17]. However, these modifications will not be discussed in this review.

Here, we will highlight metal nitrosylation, tyrosine nitration, and S-nitrosylation, discuss their importance and mechanisms of formation, and finally present recent examples of plant proteins modified by NO.

## 2. Metal Nitrosylation in Plants

Because of its chemical properties, NO can interact with transition metals of metalloproteins to form metal-nitrosyl complexes. More precisely, NO^•^ binds iron, zinc or copper centers of metalloproteins through coordination chemistry [18]. The bound NO group is then susceptible to further nucleophilic or less frequently electrophilic attacks, depending on the protein bounded [18,19]. This reactivity explains the possible involvement of metal-nitrosyl complexes in the formation of *S*-nitrosothiols groups [20]. The reversible formation of the metal-nitrosyl complex will induce conformational changes that impact the reactivity or the activity of the concerned target proteins [18,19]. This PTM has been highlighted through the use of different analytical techniques such as infrared spectroscopy, electron paramagnetic resonance, or crystallography assays [21–23].

In animals, a well-described model for this kind of PTM is the activation of soluble guanylate cyclase (sGC) after *N*-methyl-d-aspartate (NMDA) receptor activation in neurons. NO binds to the six-coordinate complex heme of sGC, which rapidly is converted to a five-coordinate ferrous nitrosyl complex. This reaction will cause the rupture of Fe-His bound in the heme, leading to conformational changes that activate sGC (for review see [24]). This activation results in the production of cyclic guanosine 3′,5′ monophosphate (cGMP), a second messenger involved in different signaling processes [25]. In plants, cGMP has also been linked to NO-dependent signaling [4] and response to biotic and abiotic stresses, such as NaCl exposure or pathogen attack [26,27]. Although several sGC have been characterized in plants, only one recent work identified a protein from *Arabidopsis thaliana* exhibiting a sGC activity modulated by NO [28]. In this work, authors identified and characterized AtNOGC1. This protein has been identified using *in silico* analysis searching for plant proteins containing a heme-binding motif and a catalytic center of plant GC. Using recombinant protein and the *in vitro* activity test, they have shown that AtNOGC1 displays an increased GC activity when treated with NO. This work strengthens a direct link between NO and cGMP signaling, but the physiological role of this protein and its regulation by NO remain to be determined *in planta*.

The best characterized plant protein undergoing metal nitrosylation is hemoglobin. In plants, hemoglobins are separated in three groups based on their structural properties, namely class 1, 2 and truncated hemoglobin class 3 [29–31]. Class-1 nonsymbiotic hemoglobins (nsHb) have a high affinity for oxygen with a low *K_D_* and are therefore unlikely to function as oxygen transporters [29–31]. Over the past decade, extensive work has shown that, in plants, oxygenated class 1 nsHbs can be oxidized by NO, resulting in nitrate production [32–34]. This NO scavenging reaction is now accepted as a general mechanism modulating NO bioavailability, participating in the regulation and detoxification of NO in plants [2,29–31,35]. Some studies also reported similar processes for class-2 symbiotic hemoglobins (sHb) that are able to interact with NO and to scavenge it [36–39]. Few data are available for the third class of Hb and no roles of these proteins in NO signaling have yet been demonstrated in plants.

Beside this NO scavenging role of Hb, recent data have demonstrated that both class-1 and class-2 nsHb could display a nitrite reductase activity, leading to the formation of nitric oxide *in vitro* during hypoxia or anoxia [40,41]. These results reinforced the link between NO signaling and hemoglobins. Nonetheless, complementary analysis must be performed to confirm such activity *in vivo*.

Few more studies report the inhibition of some target proteins through metal nitrosylation in plants such as the ascorbate peroxidase, the cytochrome c oxidase, the lipoxygenase or the catalase [42–45]. Recently, Gupta and colleagues [46] have shown that NO produced during hypoxia inhibits the aconitase, resulting in an increased level of citrate in *A. thaliana*. Therefore, the precise mechanisms of inactivation of this protein remain to be determined.

## 3. Tyrosine Nitration in Plants

Tyrosine (Tyr) nitration is the reaction of a nitrating agent with a tyrosine residue of a target protein that lead to the addition of a nitro group (NO_2_) in the *ortho* position of the phenolic hydroxyl group, resulting in the formation of 3-nitrotyrosine [47]. The NO_2_ group originates mainly from peroxynitrite (ONOO^−^), a powerful oxidative agent resulting from the reaction between NO and the superoxide anion. Under physiological condition, ONOO^−^ can react with CO_2_ and be further decomposed in CO_3_^•−^ and NO_2_, a powerful nitrating agent. Moreover, Tyr nitration can result from the reaction of nitric oxide with tyrosyl radicals [48].

Tyr nitration is restricted to specific target tyrosine residues [49,50] and can promote conformational changes that lead to the activation or the inhibition of the target proteins. Although this modification was first thought to be irreversible, a growing body of evidence tends to demonstrate that denitration processes may occur, both in enzymatic or nonenzymatic ways [51,52]. Moreover, even if this PTM is associated with protein degradation in animals [51], it has not been proved in plants. Furthermore, some proteomics analyses done in *A. thaliana* challenged by a pathogen have demonstrated that the increase in nitrated proteins is a transient phenomenon, suggesting that it is a reversible mechanism [53,54].

The study of this PTM has been mainly based on two methods. The first one is the use of specific antibodies raised against 3-nitrotyrosine residues, allowing the immuno-purification of the modified proteins or their detection in western blotting experiments [55]. The other is the use of chemical analysis techniques such as chromatography purification before mass spectrometry analysis [56–58]. Moreover, some techniques resulting in the specific fluorescent labeling of the 3-nitrotyrosine residues have been recently developed [59].

The biological significance of this PTM is not well established in plants. It is likely that Tyr nitration interferes with signaling based on phosphorylation/dephosphorylation, especially if both modifications involve the same Tyr residues. However, no published data is yet available for such mechanism in plants [52].

Only few works have been done so far to determine Tyr nitrated proteins in plants. Early approaches reporting evidence for the occurrence of Tyr nitration in plants concerned tobacco cells invalidated for the expression of nitrate reductase [60] or treated by an elicitor [61], leaves of salt-stressed olive plants [62] and *A. thaliana* challenged by an avirulent strain of *Pseudomonas syringae*[54].

More recent studies aimed in identifying the proteins undergoing this PTM. The first work was carried out on *A. thaliana* infected by *P. syringae*[53]. In this work, authors identified eleven proteins that are undergoing Tyr nitration during the development of the hypersensitive response (HR), a form of programmed cell death (PCD) triggered by plant cells at the sites of pathogen infection. The proteins identified are related to primary metabolism, such as nitrogen assimilation, ATP synthesis, Calvin cycle and glycolysis and photosynthesis. Interestingly, another group pointed out the importance of Tyr nitration as a significant PTM for the proteins involved in the photosynthetic apparatus [63,64].

Using anti-nitrotyrosine antibodies immuno-purification coupled with mass spectrometry analysis, Chaki and colleagues [65] identified 21 nitrated proteins in total extracts from hypocotyls of untreated sunflowers plants. These proteins are involved in several processes, such as the primary metabolism (photo- and ATP synthesis, carbohydrate and nitrogen metabolism), the proteasome pathway and cell signaling and antioxidant machinery. A further work done on sunflower hypocotyl submitted to high temperatures allowed the identification of these 21 proteins plus a new one, a carbonic anhydrase [65]. Moreover the exposition of the sunflowers plants to a high temperature induced a stronger Tyr nitration of some of the previously identified nitrated proteins, whereas none of them displayed a reduced level of nitration.

A recent extensive identification of nitrated proteins in plants has been carried out on crude protein extracts from *A. thaliana* seedlings using immuno-purification followed by mass spectrometry analysis. In this study, Lozano-Juste [66] identified 127 proteins that are putatively Tyr nitrated in *A. thaliana*. Among them, 35% have homologs that were previously reported to be nitrated in other organisms, supporting the relevance of these results. Remarkably, here again more than 60% of the identified proteins are involved in the primary metabolism.

More recently, Tanou and colleagues [58] assayed the Tyr nitration content of citrus plants, both in leaves and roots, after salt stress or chemical treatment with NO or H_2_O_2_. Out of these analyses, they were able to identify 88 and 86 putative candidates in leaves and roots, respectively. Remarkably, 23% of the candidate proteins in leaves are involved in photosynthesis.

If all these recent studies provided putative candidates for Tyr nitration, only few studies characterized a single protein with the determination of the nitrated residue(s). These more detailed characterizations are essential to confirm the potential candidates obtained by the broad proteomic analyses presented above, and to confirm the biological impact of this PTM.

In their study of high temperature treated sunflowers, Chaki and colleagues [67] demonstrated that the ferredoxin-NADP oxidoreductase (FNR), an enzyme mediating the final step of photosynthetic electron flow in chloroplasts, was inhibited after SIN-1 (a peroxynitrite generator) treatment *in vitro* and after high-temperature treatment *in vivo*. Nevertheless, the precise Tyr residue involved in this mechanism remains to be determined.

Àlvarez and collegues [68] recently reported the inhibition of *O*-acetylserine(thiol)lyase A1 (OASA1) by Tyr nitration in *A. thaliana*. They have demonstrated that this protein undergoes Tyr nitration selectively on its Tyr^302^ residue *in vivo* after a treatment with SIN-1. The authors explained that inactivation of this enzyme could avoid an extra production of cysteine and/or glutathione, preventing locally the scavenging of reactive oxygen and nitrogen species, further needed in downstream signaling events for an efficient stress response.

Using the symbiotic model involving root nodule formation between *Medicago truncatula* and *Sinorhizobium meliloti*, Melo and collegues [69] have shown that one of the guanylate cyclase (MtGS1a) is modified by Tyr nitration. The nitration of the Tyr^167^ residue of this protein results in a loss of its activity and occurs *in vivo* in response to an impaired nitrogen fixation, modulating the MtGS1a activity in accordance with the cell requirement in ammonia assimilation.

## 4. *S*-Nitrosylation in Plants

*S*-nitrosylation, also known as *S*-nitrosation, constitutes the most studied and described NO-dependent PTM in plants. It refers to the reversible covalent binding of an NO moiety to the thiol group of a cysteinyl residue (Cys) of a target protein, leading to the formation of an *S*-nitrosothiol (SNO; [70]). Depending on the target protein concerned, this PTM will lead to a modification of its enzymatic activity or its protein function.

NO can exist in three different reactive states—nitrosonium cation (NO^+^), nitric oxide radical (NO^•^), and nitroxyl anion (NO^−^)—which show different reactivities with thiol groups. While NO^•^ do not directly interact with thiols, NO^+^ confers strong electrophilicity and reactivity towards most biological R-SH species. However, besides the direct *S*-nitrosylation activity of NO, this molecule mainly functions as a precursor for several higher nitrogen oxides, which effectively mediate *S*-nitrosylation of proteins. Additionally, *S*-nitrosylation can also be achieved through the exchange of the NO moiety from an *S*-nitrosylated protein in a so-called transnitrosylation reaction [71,72]. Besides the possibility of the existence of other proteins with suspected transnitrosylases activity in animals [20], GSNO appears to be one of the major actors of the transnitrosylase activity in plants, modulating the total SNO content [72–75].

Despite the fact that Cys is present in the majority of plant proteins, *S*-nitrosylation is restricted to specific Cys residues. This specificity seems to be driven by the presence of surrounding acidic and basic amino acids in the vicinity of the considered Cys, and the presence of this residue in an hydrophobic pocket that can favor the concentration of nitrosylating agents [20]. Moreover, allosteric and conformational mechanisms that increase the nucleophilicity of the Cys residue favor the *S*-nitrosylation, as well as a colocalization of the production of nitrosylating agents with their target proteins [76]. However, the specificity of *S*-nitrosylation is still up for discussion. A recent analysis of 55 known S-nitrosylated proteins containing 70 NO-Cys sites revealed that proximal acid–base motif, Cys pKa, sulfur atom exposure, and Cys conservation or hydrophobicity in the vicinity of the modified Cys, do not define the specificity of *S*-nitrosylation. Instead, this analysis revealed a revised acid–base motif, which is located more distantly to the Cys and has its charged groups exposed [77].

As a major PTM, *S*-nitrosylation is a reversible and dynamic mechanism. Nonenzymatic and enzymatic ways have been proposed to promote denitrosylation of target proteins and tightly regulate this Cys modification [17,70,78,79].

The study of the *S*-nitrosylated proteins has been mainly based on the use of the biotin switch, a pioneer technique developed by Jaffrey and colleagues [80]. This technique refers to the labeling of *S*-nitrosylated proteins, allowing in a three step reaction the replacement of the SNO group by a biotin tag. This specific labeling allows the further purification and/or detection of *S*-nitrosylated proteins using affinity chromatography or antibodies detection techniques, and can be coupled with mass spectrometry analysis for further identification [70,81]. Based on this specific labeling of *S*-nitrosylated proteins, several other proteomic-based approaches have been recently developed (for review see [20,70]).

Study of *S*-nitrosylation in plants is a recent topic of interest, but over the last seven years, an increasing amount of analyses and characterizations provided evidences that it is a major PTM in plants. The first analyses were based on proteome wide-scale analysis in order to identify potential candidate proteins undergoing *S*-nitrosylation in plants. Analyses were done on *A. thaliana* plants untreated or after a brief salt stress [81], *A. thaliana* seedlings exposed to NO gas [82], to *P. syringae* pv. *tomato*[83], in the medicinal plant *Kalanchoe pinnata* after GSNO treatment of protein extracts [84] and in *Brassica juncea* exposed to low temperature [85], in citrus exposed to salinity, H_2_O_2_ or GSNO treatments [58,86] and more recently in rice *noe1* (*nitric oxide excess1*) mutant [87] and in tobacco cell suspensions exposed to the oomycete elicitor cryptogein [88]. Protein *S*-nitrosylation was also investigated in organelles, such as purified mitochondria of *A. thaliana*[89] and peroxisomes of pea plants exposed to abiotic stress [90]. All these analysis provide a list of over 200 putatively *S*-nitrosylated plant proteins. However, for the most part of them, *S*-nitrosylation were obtained using pharmacological NO donors. Moreover, the *S*-nitrosylation of these candidates needs confirmation by a candidate-specific approach, to ensure a mechanistic and biological comprehension of the impact of the *S*-nitrosylation in plants.

Apart from these general proteomic approaches, around 20 different candidate proteins have been more tightly characterized [17,33,54,84,88,89,91–99]. The functional significance of the *S*-nitrosylation of these candidates has been recently reviewed for the most part of them [70,75,100].

Interestingly, most of the characterized proteins are linked or potentially linked to the plant immunity. Peroxiredoxin II E (PrxII E) belongs to the peroxiredoxin family that detoxifies a large set of peroxide substrate and participates in redox signaling in plants. Among them, PrxII E displays an ONOO^−^ reductase activity [54] that is inhibited through the *S*-nitrosylation of its Cys^121^ residue in *A. thaliana* challenged with *P. syringae* bacteria [54,83]. This phenomenon is proposed by the authors as a mechanism allowing a fine tuning of the NO signaling, avoiding damaging and signaling effect of ONOO^−^ notably through Tyr nitration.

Another plant signaling pathway leading to defense responses and impacted by NO through *S*-nitrosylation involves the nonexpressor of pathogenesis-related gene 1 (NPR1)/Transcription factor TGA1 system. In plants, NPR1 is a key regulator of salicylic acid (SA)-dependent signaling that promotes defense responses in plants. Following oxidative changes triggered by SA, NPR1 hexamers dissociate through the reduction of intermolecular disulfide bounds into monomers, and is translocated into the nucleus where it interacts with TGA factor including TGA1, allowing the expression of defense related genes [101]. Therefore, the oligomer/monomer switch and the NPR1/TGA interaction are critical for triggering plant defense responses. Despite the existence of contradictory data, *S*-nitrosylation of these proteins has been shown to be crucial in the regulation of theses processes (for review see [70,75,100]). Another protein linked to SA signaling pathway has also been characterized: the salicylic acid binding protein 3 (SABP3). SABP3 possesses carbonic anhydrase (CA) activity and SA binding properties and is involved in the development of the HR in tobacco [102]. In *A. thaliana* challenged by an avirulent *P. syringae* strain, SABP3 undergoes *S*-nitrosylation on its Cys^280^ residue that leads to the decrease of the CA activity and SA binding properties of the protein [97]. All these data highlight the connection between SA and NO signaling in the establishment of plant defense responses.

One recent important work allowing a better comprehension of the NO signaling in plants and involving *S*-nitrosylation concerns the NADPH oxidase [98]. AtRBOHD is a NAPDH oxidase that is responsible for reactive oxygen species (ROS) synthesis in responses to several pathogen attacks [103]. After *P. syringae* infection of *A. thaliana* plants, Yun and colleagues [98] reported the *S*-nitrosylation of AtRBOHD on its Cys^890^ residue. This PTM avoid the fixation of one cofactor of the enzyme, resulting in a decreased activity of the protein. Therefore, regulating AtRBOHD by *S*-nitrosylation NO might modulate the ROS production after a pathogen attack, which impacts the development of HR after pathogen infection.

Another recent work identified physiologically *S*-nitrosylated candidates in a plant defense context. Indeed, the S-nitrosylation of eleven proteins has been reported subsequently to the recognition of the oomycete elicitor cryptogein [88], known to trigger a fast and transient NO production in tobacco leaves and cell suspensions [104–106]. Among these proteins, NtCDC48 has been further characterized. CDC48 (cell division cycle 48) is a hexameric AAA+ ATPases (ATPases associated with various cellular activities) involved in multiple cellular pathways, including growth, development, cell division and differentiation, protein degradation and disease resistance [107–110]. NtCDC48 has been shown to be *S*-nitrosylated on its Cys^526^ residue, which is required for the full activity of the protein *in vitro*. NO donor treatments of the recombinant protein resulted in subtle conformational changes and a decrease of the activity of NtCDC48 *in vitro*. Nevertheless, further investigation must be carried out to decipher the impact of NtCDC48 *S*-nitrosylation in plant defense reaction triggered by cryptogein *in planta*.

Recently, it was demonstrated that the transport inhibitor response 1 (TIR1) protein of *A. thaliana* undergoes *S*-nitrosylation *in vitro*[96]. TIR1 is a receptor subunit for auxin, part of an E3-ubiquitin ligase complex involved in the degradation of transcriptional repressors called Auxin/indole-3acetic proteins (Aux/IAA). This degradation results in the transcription of genes and participates to the auxin signaling, involved in plant development. Interestingly, *S*-nitrosylation of TIR1 promotes its interaction with Aux/IAA proteins, facilitating their degradation, and therefore taking part in the auxin-dependent signaling pathway [96]. This example illustrates the involvement of the *S*-nitrosylation, not only in plant defense, but also in plant development.

## 5. Conclusions

**Figure 1:**
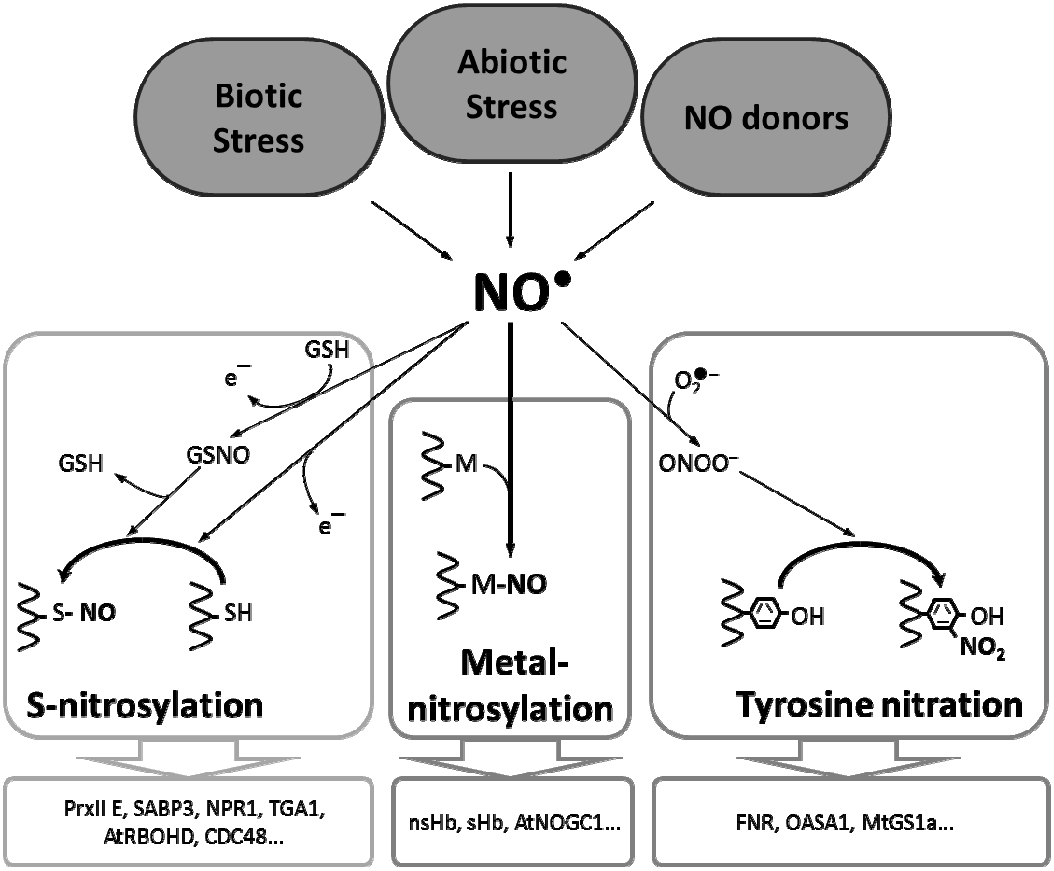
Schematic illustration of NO dependent PTM in plants. To date, all the analyses of NO-modified proteins in plants followed an NO production induced by (a)biotic stress or NO donors treatment. The NO radical can react with transition metals (M) of metalloproteins. This process is called metal nitrosylation and can affect notably (non)-symbiotic hemoglobins (nsHb and sHb) and an *Arabidopsis thaliana* NO-dependent guanylate cyclase (AtNOGC1). The Tyr nitration depends on the formation of NO derivatives, particularly peroxynitrite formed in the presence of the superoxide anion (O2^•−^). Nitration occurs on one of the two carbon equivalent (C3) of the aromatic ring of tyrosine residues to form a 3-nitrotyrosine. This reaction has been demonstrated in plants for the ferredoxin-NADP oxidoreductase (FNR), the guanylate cyclase of *Medicago truncatula* (MtGS1a) or the of O-acetylserine(thiol)lyase A1 (OASA1). Protein *S*-nitrosylation is the electrophilic attack of nitrosonium cation (NO^+^, resulting from the oxidation of NO) on a thiolate group of a cysteine residue of a target protein. Among numerous proteins, this posttranslational modification affect for example peroxyredoxin II E (PrxII E), salicylic acid binding protein 3 (SABP3), nonexpressor of pathogenesis-related gene 1 (NPR1), transcription factor TGA1, respiratory burst oxidase homologue D (RBOHD) or cell division cycle 48 (CDC48). All these modifications will participate to the change of the plant cell physiology depending on the stimulus applied.

NO-triggered PTMs constitute today a major field of investigation in order to better understand the NO-dependent signalization in plants (Table 1, Figure 1). To date, metal nitrosylation reports in plants are scarce, and the main purpose of this PTM is assumed to be a scavenging and detoxification process. More effort must be put on determining the involvement of this PTM in the induction of further signaling events, such as the production of cGMP by putative NO-dependent sGC. On the contrary, Tyr nitration has begun to be recognized over the last years as an emerging and important PTM in plants. Nevertheless, a lot of work remains, such as by using more physiological approaches to decipher the real biological role of this PTM in general plant physiology, thus allowing for a better understanding of the impact of NO. *S*-nitrosylation is the best characterized NO-dependent PTM and a general picture of the involvement of this PTM is emerging in different processes, especially in plant defense mechanisms against biotic and abiotic stresses. Here again, several efforts are needed to better understand the relevance of this PTM in physiological contexts.

## Figures and Tables

**Table 1:** Examples of nitric oxide target proteins in plants.

Posttranslational modification	Target protein	References
Metal nitrosylation	NO-dependant Gunylate cyclase 1 (AtNOGC1)	[24]
	Hemoglobins	[25–37]
	Aconitase	[42]
Tyrosine nitration	Ferredoxin-NADP oxidoreductase (FNR)	[63]
	O-acetylserine(thiol)lyase A1 (OASA1)	[64]
	Guanylate cyclase	[65]
*S*-nitrosylation	Peroxiredoxin II E (PrxII E)	[50]
	Nonexpressor of pathogenesis-related gene 1 (NPR1)	[75,90]
	Transcription factor TGA1	[90]
	Salicylic acid binding protein 3 (SABP3)	[93]
	Respiratory burst oxidase homologue D (RBOHD)	[94]
	Cell division cycle 48 (CDC48)	[84]
	Transport inhibitor response 1 (TIR1)	[92]
